# Edward Harrison (1759–1838): An overlooked advocate of EcoHealth and One Health in the early 19th century

**DOI:** 10.1177/0967772020921668

**Published:** 2020-04-28

**Authors:** Neil J Morley

**Affiliations:** School of Biological Sciences, Royal Holloway, University of London, Egham, Surrey, UK

**Keywords:** Liver fluke, malaria, tuberculosis, *Fasciola hepatica*, epidemiology, EcoHealth, One Health

## Abstract

Edward Harrison (1759–1838) was an English doctor best known for his ground-breaking treatments of spinal deformities and his failed attempts at medical reforms in the early 19th century. However, with the encouragement of his patient and patron Sir Joseph Banks, he also undertook comparative research on the influence of environmental factors on infectious diseases of medical and veterinary importance using approaches that were forerunners of the modern-day concepts of EcoHealth and One Health. His works in this field, particularly his study of sheep rot, are highlighted.

Present-day understanding of the historical development of integrated research on important medical and veterinary infectious diseases under the influence of environmental factors is dominated by a few individuals who were mainly active from the mid-19th century onwards. Modern-day researchers term this field ‘Ecohealth’ or ‘One Health’, two broad but related concepts that characterise integrated efforts to address associated problems of the health of humans, animals, and ecosystems. However, the pre-modern era of the early 19th century has received less attention and only a handful of researchers are generally noted.^1,2^ This period saw the emergence of medical geography with many individuals developing an ecosystem-approach to human health and infectious disease aetiology yet those envisaging links with animal diseases were much fewer, the most prominent being the French physician Felix Vicq d’Azyr.^2^

One such overlooked researcher is Edward Harrison (1759–1838), an English doctor mainly known for his attempts at medical reforms and his ground-breaking work on the treatment of spinal deformities.^3^ However, he also undertook influential research on the relationship between ecosystems and comparative infectious diseases of humans and animals using integrated approaches that were forerunners of modern-day concepts.

Harrison had a busy provincial medical practice in the town of Horncastle, Lincolnshire. This area was on the edge of the Fens, a large sprawling marshland in the east of England. Outside of the main roads and towns the land was largely inaccessible, often fog-bound, and regarded as an unhealthy environment with high-levels of mortality. It was a region where the ague, or malaria, was particularly prevalent,^4^ such that the landscape played a key role in the lives and health of the residents and was influential in shaping Harrison’s understanding of diseases. Within his practice, his most distinguished patient was Sir Joseph Banks, whom he treated for gout.^3^ Banks was not only a well-respected naturalist and President of the Royal Society but also a wealthy Lincolnshire landowner who took a keen interest in the well-being of the area^5^ and acted as a patron for some of Harrison’s early scientific investigations.^3^

Harrison’s geographically diverse practice gave ample opportunity to observe how the environment where individual patients resided influenced their health. Over many years he noticed that pulmonary diseases, particularly tuberculosis, were uncommon amongst residents of the fens and marshes when compared with other non-marshy districts.^6^ Although a number of different theories on the aetiology of diseases were available at this time, Harrison’s interpretation of the causes of his observations was based on the miasmic theory of disease, a widely-accepted belief that most infections were the result of inhaling air that had become contaminated through exposure to corrupting matter such as rotting corpses, vegetation, or sewage.^7^ Influenced by the theories of Samuel Latham Mitchill, that disease-causing nitrous miasmas could be absorbed by lime and therefore the healthiest regions were where calcareous outcrops and soils were common,^8^ Harrison considered similar variations in the Lincolnshire landscape as being a key influence on miasma occurrence and in turn the epidemiology of diseases such as tuberculosis and malaria in the county.

Harrison also noted the similarities between human and sheep tuberculosis and suggested these animals may be useful comparative subjects for experimentally determining the importance of environmental conditions in the development of pulmonary diseases.^6^ Over time Harrison’s interest in sheep and their diseases grew, focusing particularly on the devastating condition known as ‘rot’. However, although this interest was largely inadvertent his approach was always based on the assumption that these animals may throw new light on the diseases of humans.^9^

Sheep rot is a disease now known to be caused by the zoonotic liver fluke *Fasciola hepatica* whose incidence is linked to environmental conditions that favour the occurrence of the semi-aquatic snail intermediate host.^10^ In the early 19th century its specific causes were unknown. Nevertheless, the connection between humidity, in the form of wet pasture, and the occurrence of rot was generally acknowledged. Confirmation of sheep rot involved opening up the carcass and examining the liver which typically displayed gross pathological damage. The presence of flukes in this diseased organ was frequently, but not always, noted and it was much disputed if they were a by-product or the cause of rot.^11^ However, the deaths of millions of sheep during periodic epizootics made a greater understanding of the disease of immense economic importance. In the summer of 1802 Harrison sent out a questionnaire to landowners and farmers on their understanding of the causes and conditions that gave rise to sheep rot.^12^ His questions principally focused on the type of soil and moisture levels of habitats where rot occurred, the symptoms and circumstances of disease occurrence, along with its potential relationship to the presence of liver fluke.

The feedback he received from this questionnaire provided a valuable overview of the levels of traditional knowledge of the disease and was used as a basis for his comparative study on the importance of environmental conditions to the occurrence of rot and potentially related human diseases such as tuberculosis and malaria. Harrison intended to complement this survey work with experimental investigations on sheep rot under the supervision of Sir Joseph Banks; however, conditions during the intended season were unsuitable and this aspect of the study was never instigated.^13^ Harrison sent his completed report to Banks, who arranged for its publication in *Annals of Agriculture*, a leading scientific journal of the period. In a covering letter published alongside the study Harrison expressed the hope that “to the medical practitioner a field of inquiry is laid open by which he will be enabled to explain and to obviate some of the most intricate and important disorders of mankind”.^13^ Inconsistent evidence on the role of the liver fluke, particularly the variable levels, or complete absence, of localized liver damage in close proximity with any noticeable worms, and the claims by certain authorities that unborn lambs in the womb could also suffer from rot without discernible flukes raised questions in Harrison’s mind as to their significance. He consequently disregarded the flukes’ involvement in sheep rot and concluded that the causes of this and other diseases were the result of certain pathogen-specific miasmas associated with different soil conditions which induced particular diseases in animals and humans.

A revised and expanded report, dedicated to Sir Joseph Banks, was published as a monograph a year later.^14^ More widely distributed, it resulted in much discussion with one commentator considering Harrison’s approach as a fresh branch of the science of comparative anatomy which they termed ‘comparative pathology’.^15^ Due to the extensive traditional knowledge presented about sheep rot Harrison’s inquiry became the definitive veterinary account of the early 19th century in the UK and formed the basis of later scientific approaches to the disease. It was regarded as the standard reference for many years^16–18^ and its continuing importance resulted in its reprinting 25 years later.^19^ The monograph’s comparative approach to animal and human diseases was still regarded at this time as enlightening with much of value for more recent scientific investigations^20^ and it remained a recommended text for British veterinarians into the 1840s.^21^ Although Harrison continued to make observations on this subject,^19^ he issued no further publications after his interests switched to more pressing concerns regarding medical reforms.^3^

Harrison is an unfairly forgotten figure in the history of integrated medical and veterinary research whose theories on infectious diseases continued to provoke debate in the UK into the mid-19th century.^22–24^ Despite his erroneous hypotheses on miasmas as the causes of diseases, his enlightened comparative approaches to understanding disease epidemiology and their relationship to environmental conditions make his contributions noteworthy in the historical development of this field.
